# Early prediction of colorectal adenoma risk: leveraging large-language model for clinical electronic medical record data

**DOI:** 10.3389/fonc.2025.1508455

**Published:** 2025-05-15

**Authors:** Xiaoyu Yang, Jinjian Xu, Hong Ji, Jun Li, Bingqing Yang, Liye Wang

**Affiliations:** ^1^ Information Management and Big Data Center, Peking University Third Hospital, Beijing, China; ^2^ Department of Gastroenterology, Peking University Third Hospital, Beijing, China; ^3^ Goodwill Hessian Health Technology Co. Ltd, Beijing, China

**Keywords:** adenoma, colorectal adenoma, large language model, early prediction, electronic medical record, colorectal cancer

## Abstract

**Objective:**

To develop a non-invasive, radiation-free model for early colorectal adenoma prediction using clinical electronic medical record (EMR) data, addressing limitations in current diagnostic approaches for large-scale screening.

**Design:**

Retrospective analysis utilized 92,681 cases with EMR, spanning from 2012 to 2022, as the training cohort. Testing was performed on an independent test cohort of 19,265 cases from 2023. Several classical machine learning algorithms were applied in combination with the BGE-M3 large-language model (LLM) for enhanced semantic feature extraction. Area under the receiver operating characteristic curve (AUC) is the major metric for evaluating model performance. The Shapley additive explanations (SHAP) method was employed to identify the most influential risk factors.

**Results:**

XGBoost algorithm, integrated with BGE-M3, demonstrated superior performance (AUC = 0.9847) in the validation cohort. Notably, when applied to the independent test cohort, XGBoost maintained its strong predictive ability with an AUC of 0.9839 and an average advance prediction time of 6.88 hours, underscoring the effectiveness of the BGE-M3 model. The SHAP analysis further identified 16 high-impact risk factors, highlighting the interplay of genetic, lifestyle, and environmental influences on colorectal adenoma risk.

**Conclusion:**

This study developed a robust machine learning-based model for colorectal adenoma risk prediction, leveraging clinical EMR and LLM. The proposed model demonstrates high predictive accuracy and has the potential to enhance early detection, making it well-suited for large-scale screening programs. By facilitating early identification of individuals at risk, this approach may contribute to reducing the incidence and mortality associated with colorectal cancer.

## 1 Introduction

Colorectal adenoma, the primary precancerous lesion in colorectal cancer (1), poses a significant health concern. It is estimated that 70-90% of colorectal cancers originate from these adenomas, underscoring the critical importance of early risk prediction and screening (2–4). However, current screening methods, including fecal examination, endoscopy, and CT colonography (5, 6), have limitations that hinder their widespread application. Fecal examination, while preferred for its comfort and safety, has limited specificity. Endoscopy, the gold standard, is invasive, technically demanding, and requires bowel preparation, limiting its application for large-scale screening (7). CT colonography, a non-invasive and highly sensitive alternative, necessitates specialized equipment, technical expertise, and rigorous patient preparation, along with radiation exposure (8).

To address these challenges, we proposed an innovative approach that leverages the power of machine learning and electronic medical record (EMR) data for accurate and widespread colorectal adenoma prediction. Machine learning techniques have shown promising results in identifying adenomas from colonoscopy or pathology images (9–11). Additionally, researchers have identified significant risk factors, such as smoking, alcohol consumption, and obesity-related indicators, through various analyses (12–14). However, existing studies lack individualized risk classification, limiting their clinical application.

EMR data presents a valuable and suitable alternative for large-scale screening, and researchers have developed risk prediction models to guide high-risk patients towards colonoscopy, optimizing resource allocation (15–20). Nevertheless, previous studies have been limited by small sample sizes and insufficient EMR data. Traditional One-Hot coding of EMR data fails to capture semantic information and is sensitive to missing data. In contrast, recently popular pre-trained large language models (LLMs) have emerged as a promising solution, demonstrating superior performance in extracting and encoding semantic information for disease diagnosis and prognosis (21–23).

Building upon these advancements, this study leverages comprehensive EMR data, including medical history, symptoms, tests, and examinations. By utilizing a semantic vector model based on the BGE-M3 coding approach, we aim to improve data comprehension, reduce sparsity, and improve generalization. This study aims to develop a robust risk prediction model for colorectal adenoma, providing real-time risk assessments to support informed clinical decision-making and ultimately improve patient outcomes.

## 2 Materials and methods

### 2.1 Dataset

**Figure 1:**
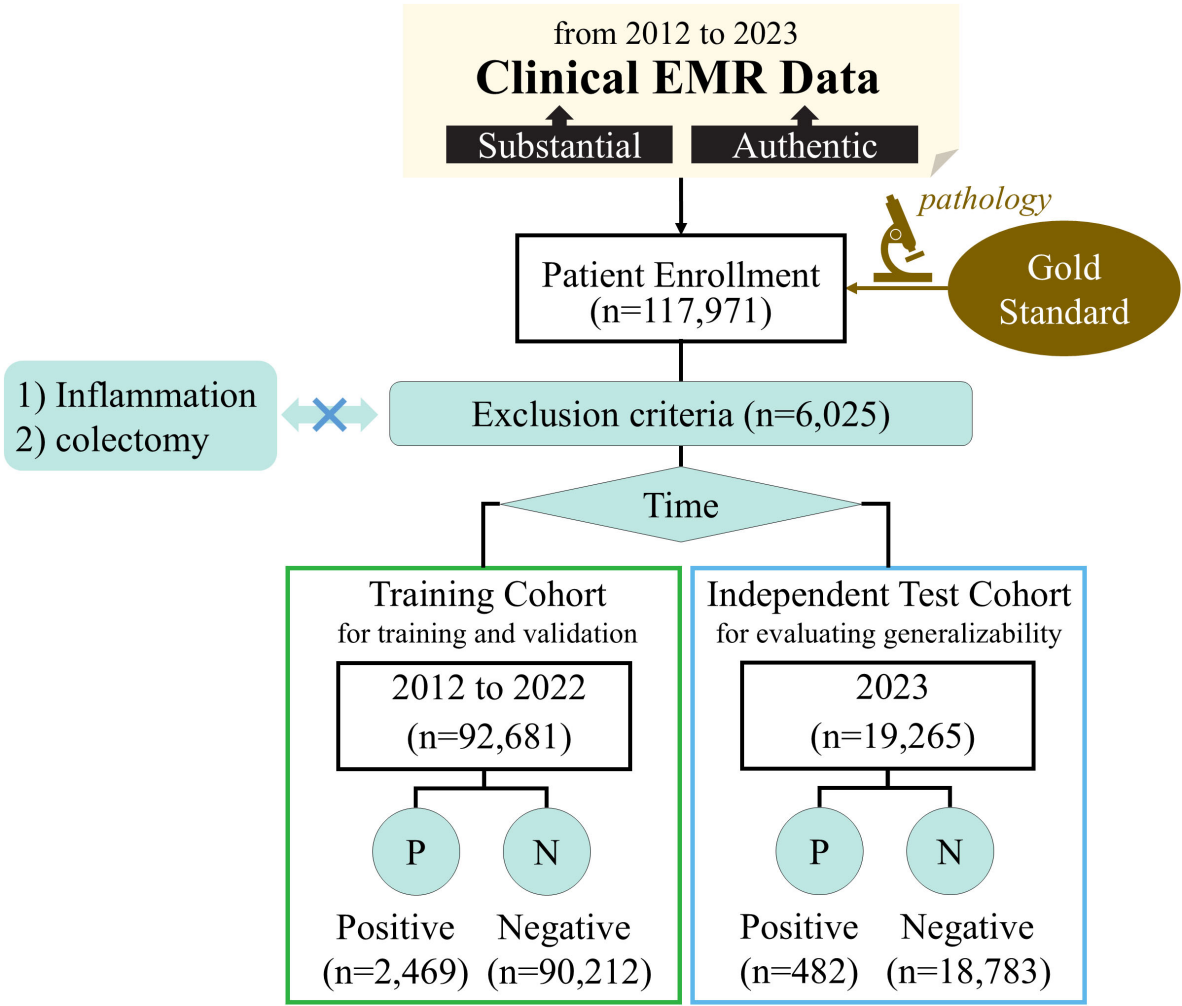
Patient enrollment for colorectal adenoma.

This retrospective study utilized EMR data from January 2012 to December 2023 at a high-performing hospital with an annual outpatient volume of approximately 4.5 million. The study was approved by the Medical Science Research Ethics Committee of **** hospital, and the requirement for patient informed consent was waived due to the retrospective nature of this study. The data collection process yielded a total of 2,951 positive and 108,995 negative samples. **Figure 1** provides a detailed overview of the enrollment and data collection process.

#### 2.1.1 Data inclusion and exclusion

Inclusion criteria for the study were as follows: (1) patients aged 18 years or older at their initial visit; (2) pathology reports confirming the presence of colorectal adenoma; (3) visits to the gastroenterology department with relevant diagnoses or pathology reports excluding colorectal adenoma.

Exclusion criteria were defined as follows: (1) patients with a history of colectomy or partial colectomy; (2) medical histories or current complaints involving conditions that could influence inflammation assessment, including but not limited to colorectal adenoma, colorectal cancer, colon cancer, cecum cancer, rectal cancer, rectosigmoid junction cancer, inflammatory bowel disease, diverticulitis, cirrhosis of the liver, abnormal liver function, acute pancreatitis, pancreatic cancer, neuroendocrine tumor, capillary dilatation, lipoma, jaundice.

#### 2.1.2 Data splitting

The data from 2012 to 2022 was divided into the training cohort, consisting of a total of 2,469 positive samples and 90,212 negative samples. More recent data from 2023 was reserved as an independent test cohort, comprising 482 positive samples and 18,783 negative samples. The training cohort was utilized for model development and hyperparameter tuning using five-fold cross-validation, and the independent test cohort served to evaluate the generalizability of the developed model.

#### 2.1.3 EMR feature inclusion

Clinical EMR data (**Table 1**) comprises both structured and unstructured formats. Structured data, directly extracted from the EMR, included demographic information such as gender, age and laboratory results. Laboratory items analyzed in this study encompassed glycated hemoglobin, total cholesterol, fecal occult blood test, complete blood count, biochemical profile, and tumor markers.

**Table 1:** Sources and content of feature extraction.

Type	Sources	Content
Structured Data	Medical Records Homepage	Gender, Age, etc.
	Laboratory Items	All abnormal test data prior to the diagnosis of colorectal adenoma, including but not limited to Glycated Hemoglobin, Total Cholesterol, Fecal Occult Blood Test, Complete Blood Count, Biochemical Profile, Tumor Markers, etc.
Unstructured Data	Chief Complaint/History of Present Illness (HPI)	Hematochezia, Abnormal Stool, Colonic Polyps, Abdominal Pain, Acid Reflux, Diarrhea, etc.
	Family History	Colorectal Cancer, etc.
	Physical Examination	Digital Rectal Exam, BMI, Systolic Blood Pressure, etc.
	Past Medical History	Hypertension, Hyperlipidemia, Diabetes, Metabolic Syndrome, History of H. pylori Infection, etc.
	Examination Items	Abdominal Ultrasound, Abdominal CT, etc.

Unstructured data, such as the chief complaint, history of present illness (HPI), and family history, required initial information extraction followed by content extraction to transform them into a structured format. In this paper, a multilevel entity-relationship extraction scheme was implemented to process free text data in EMR, consisting of two key steps: (1) we distinguished medical record instruments and chapters by “instrument category prediction” and “chapter prediction”; (2) we applied a Bidirectional Long Short-Term Memory-Conditional Random Field (BiLSTM-CRF) network combined with rule matching – a method that has been demonstrated as an effective approach for extracting information from unstructured data (24, 25) - to extract the information from the Chinese clinical report. These steps guarantee the extraction of clinically relevant information essential for subsequent predictive modeling. For this study, we utilized the earliest available laboratory and examination data prior to the diagnosis of colorectal adenoma. Meanwhile, we excluded the pathological examination items to prevent potential data leakage during the model development process.

#### 2.1.4 Entity unification

To ensure feature consistency, entity unification was implemented on the raw clinical records, with data quality control assured by semantic validation protocols. To avoid the various descriptions of entities such as signs, symptoms, and disease names among physicians, we utilized the British Medical Journal (BMJ) Best Practice knowledge base (26). This resource unifies different aliases of each entity to a uniform name. Subsequently, these diverse descriptions are mapped to their standardized entities, effectively reducing data fragmentation caused by alias discrepancies. For example, terms like “dry stools” and “increased bowel movements” were unified under the category of “abnormal stools”. Additionally, features with a missing rate of ≥99% were excluded to mitigate the risks of model overfitting and to improve the stability and accuracy of the model.

The examination items within unstructured data predominantly comprise imaging studies, whose non-textual characteristics hinder structured dataset conversion. Therefore, this study restricted analysis to text-based components of examination reports, specifically the “examination observation” and “examination conclusion”. In order to ensure the objectivity of the results of the study, exclusion criteria were established: (1) Examination items directly related to the pathological diagnosis of colorectal adenomas (including but not limited to colonoscopy, pathological analysis, and tissue biopsy) were excluded; (2) Examination items retained were further excluded from the records whose text descriptions explicitly contained diagnostic information of colorectal adenomas.

### 2.2 Prediction model development

**Figure 2:**
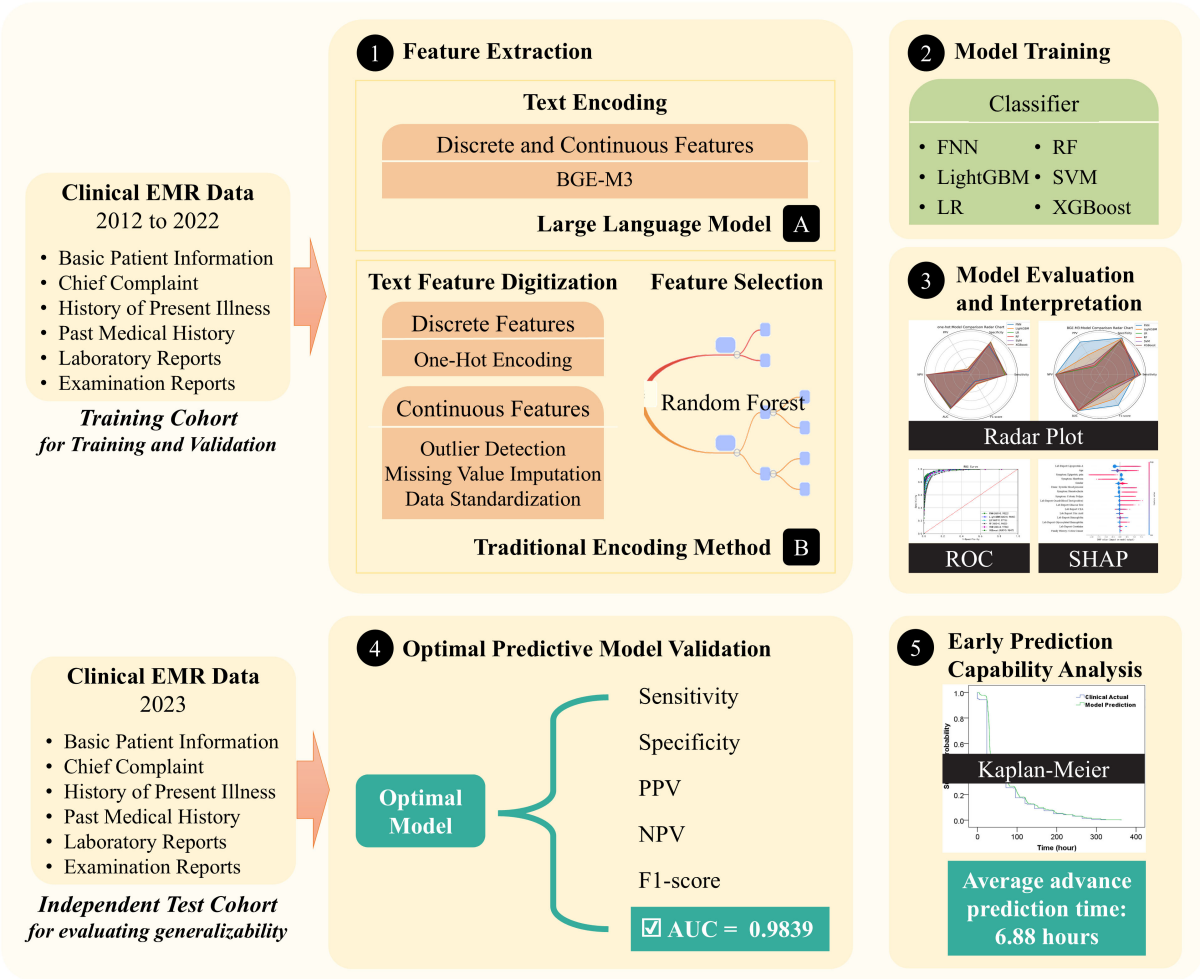
Process of developing the risk prediction model for colorectal adenoma.

The development of the colorectal adenoma risk prediction model is depicted in **Figure 2**. This process begins with the collection of comprehensive EMR data, including basic patient information, chief complaints, current and past medical histories, examination reports, and laboratory test results. During the feature extraction stage, we employed the BGE-M3 semantic vector encoding and traditional one-hot vector encoding for comparison.

For model training, several widely used machine learning models for binary classification tasks are employed, including logistic regression, random forest, support vector machine (SVM), LightGBM, XGBoost, and feed-forward neural networks. To optimize model performance, we utilized cross-validation and grid search on the training and validation cohort to fine-tune model parameters and achieve optimal predictive accuracy.

### 2.3 Feature extraction

#### 2.3.1 One-hot vector coding

One-hot encoding is a traditional technique used to represent categorical features in machine learning algorithms. Each category is converted into a binary feature for model integration. For continuous features, outlier handling methods are applied depend on the distribution of the data. Normal distribution data is evaluated using the 3σ criterion, while non-normal distribution data uses the interquartile range (IQR) criterion for outlier identification. The detected outliers are removed, and missing values are imputed with the mean to maintain data integrity. Feature scaling is then performed by dividing each value by the maximum absolute value in the feature, standardizing the feature values within the range of (–1, 1). This scaling benefits to model stability.

However, one-hot encoding can generate high-dimensional feature vectors, especially with our large EMR data, and not all features are pertinent to the prediction task. To address this, feature selection is crucial to reducing dimensionality, enhancing model efficiency, and mitigating overfitting. Random Forest, a robust machine learning algorithm, provides an effective feature selection method. It measures the importance of each feature by assessing its contribution to the reduction of Gini impurity in decision tree nodes. Features with higher importance scores are retained, streamlining data dimensionality and boosting computational efficiency.

#### 2.3.2 BGE-M3 vector coding

The Smart Source General Semantic Vector Model BGE-M3 (BAAI General Embedding M3), introduced in 2024 (27), offers significant advantages over traditional One-Hot Encoding, particularly for analyzing clinical EMR data with diverse semantic information. BGE-M3 simplifies preprocessing and effectively captures insightful semantic features, enhancing the understanding of complex clinical report, which is crucial in clinical diagnosis. It integrates semantic, syntactic, and knowledge graph data to provide detailed representation of sentence semantics. This comprehensive approach improves the accuracy and richness of sentence vectors, addressing the challenge of using complex clinical report.

BGE-M3 incorporates advanced attention mechanisms and context modeling techniques, dynamically adjusting word importance and capturing contextual information. These features improve the precision of feature vector for representing sentences, making BGE-M3 effective for extracting meaningful insights from clinical report. Additionally, BGE-M3’s optimized structure and parameter tuning ensure high computational efficiency and scalability, facilitating rapid and accurate sentence representations for handling large-scale clinical datasets.

### 2.4 Model training

Colorectal adenoma risk prediction involves a binary classification task. This study evaluates the effectiveness of several machine learning algorithms, including the feedforward neural network (FNN) (28), Light Gradient Boosting Machine (LightGBM) (29), Logistic Regression (LR) (30), Random Forest (RF) (31), Support Vector Machine (SVM) (32), and eXtreme Gradient Boosting (XGBoost) (33). Further details on the algorithms and their implementations can be found in the Section 2.4 of the Supplementary Material.

Optimal model parameters were selected through a systematic process involving grid search and five-fold cross-validation. This method entailed exploring predefined parameter ranges and randomly combining parameters across various model algorithms. Detailed descriptions of the optimal parameters and their respective ranges throughout the tuning process are provided in **Supplementary Table S1** of the **Supplementary Material**.

### 2.5 Model evaluation

#### 2.5.1 Evaluation metrics

The performance of the risk prediction model was evaluated using multiple metrics in both the validation cohort and the independent test cohort, including Sensitivity/Recall, Specificity, Positive Predictive Value (PPV), Negative Predictive Value (NPV), F1-Score, AUC, and Receiver Operating Characteristic (ROC) analysis. Detailed descriptions of the evaluation metrics are provided in **Supplementary Table S2** of the **Supplementary Material**.

#### 2.5.2 Evaluation on the independent test cohort

We evaluated the model’s ability to predict colorectal adenoma in advance on the independent test cohort. Starting from patient admission, the model predicted risk based on changes in EMR data, including health status or new laboratory test results. Predictions continued until a high risk of colorectal adenoma was indicated, and subsequent pathology diagnosis was used to verify the accuracy of the predictions. Successful predictions were defined as those matching the actual outcomes. To quantify predictive lead time, we computed the average interval between the model’s high-risk prediction and the actual diagnosis for successful predictions.

We employed Kaplan-Meier analysis to demonstrate the change in model-predicted outcomes and clinical actual diagnoses with increasing time of admission. The horizontal coordinate is the time of admission, the vertical coordinate survival rate indicates the ratio of positive samples remained incorrectly diagnosed, which means 0% indicates that all positive samples were correctly diagnosed. This method provides a graphical representation of predictive accuracy and effectiveness over time.

### 2.6 Model interpretation

We utilized the SHAP (Shapley Additive exPlanations) method to interpret the relationship between features and model predictions (34). SHAP employs an additive feature attribution approach to calculate values for each feature, quantifying both the magnitude and direction of their impact on model predictions. The absolute value of each feature’s SHAP score indicates the degree of influence on the model’s predictions. Positive SHAP values suggest support for a higher risk prediction of colorectal adenoma, while negative values indicate the opposite. This method provides insight into the model’s decision-making process and offers opportunities for further model optimization.

### 2.7 Statistical analysis

In this study, we employed TableOne, a powerful R language package, for comprehensive statistical analysis (35). Specific statistical methods were selected based on the distributional properties of the variables. For normally distributed continuous variables, quantitative results are presented as mean ± standard deviation (SD), with significance assessed using the t-test. For non-normally distributed continuous variables, the data was reported as median (Q1, Q3), and differences between groups were evaluated using the Mann-Whitney U test, a non-parametric alternative. Categorical variables were analyzed using the chi-square test to determine significant differences between categorical levels.

### 2.8 Patient and public involvement

This study was a retrospective analysis, thus patients were not involved. We have provided relevant content following the TRIPOD reporting guidelines (36).

## 3 Results

### 3.1 Data distribution of different departments

**Figure 3:**
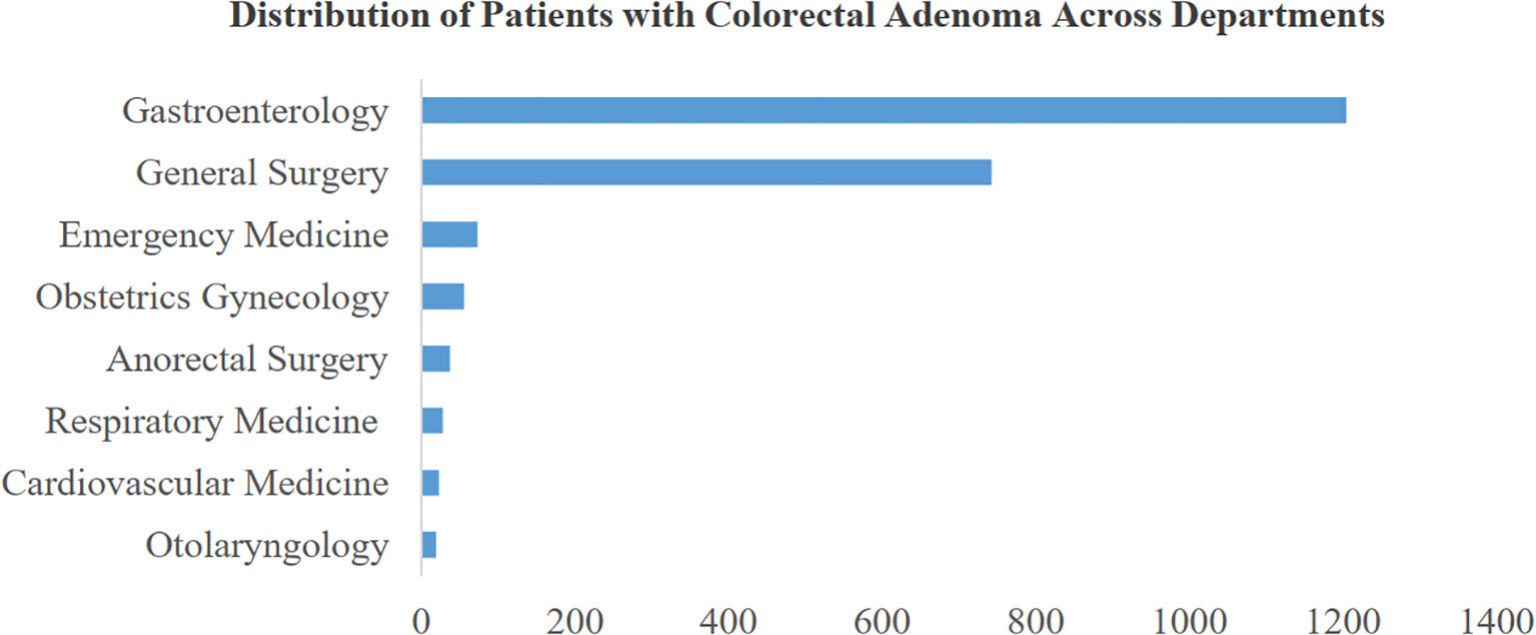
Distribution of patients with colorectal adenoma across departments.

This study examines the distribution of colorectal adenoma patients across various departments at their initial consultation. **Figure 3** illustrates the distribution of the top 8 departments based on patient numbers. Notably, the gastroenterology department exhibits a high prevalence, with approximately 1,200 cases of colorectal adenoma, indicating a significantly higher incidence compared to other departments. Interestingly, the general surgery department also demonstrates a substantial proportion of cases, suggesting that colorectal adenoma is prevalent among patients seeking care in non-gastroenterology departments. This observation underscores the relevance and potential impact of the risk prediction model proposed in this study. By extending beyond gastroenterology, our model aims to facilitate screening and preventive strategies across multiple departments. Early identification of colorectal adenoma risks can contribute to reducing the incidence of colorectal cancer and enhancing comprehensive patient care and safety.

### 3.2 Clinical characteristics analysis

Statistical analysis was performed on the clinical characteristics of patients enrolled between 2012 and 2022, as shown in **Table 2**. Since there are so many characteristics in this study, we only present those that are statistically different and clinically important. Within the present characteristics, Cholesterol, Triglycerides and CA19–9 did not show statistical differences between groups.

**Table 2:** Statistical analysis of clinical characteristics.

Feature name	Total sample size (N=92,681)	Non-colorectal adenoma group (N=90,212)	Colorectal adenoma group (N=2,469)	P-value
Demographic characteristics				
Age, mean(SD)	49.1 (15.7)	48.8 (15.7)	59.4 (14.7)	<0.05
Male, n (%)	42765 (46.1)	41407 (45.9)	1358 (55.0)	<0.05
Signs and symptoms				
Blood in stool, n (%)	5155 (5.6)	4691 (5.2)	464 (18.8)	<0.05
Nausea, n (%)	7692 (8.3)	7578 (8.4)	114 (4.6)	<0.05
Abdominal pain, n (%)	12876 (13.9)	12449 (13.8)	427 (17.3)	<0.05
Stool abnormalities, n (%)	32319 (34.9)	31304 (34.7)	1015 (41.1)	<0.05
family history				
Colon cancer, n (%)	583 (0.6)	541 (0.6)	42 (1.7)	<0.05
Colon polyps, n (%)	4465 (4.8)	4060 (4.5)	405 (16.4)	<0.05
Hypertension, n (%)	9108 (9.8)	8570 (9.5)	538 (21.8)	<0.05
Diabetes, n (%)	4006 (4.3)	3789 (4.2)	217 (8.8)	<0.05
Inspection Indicators				
Hemoglobin (g/L, mean(SD))	140.8 (15.7)	140.9 (15.7)	136.9 (16.6)	<0.05
Fecal occult blood test, n (%)	3817 (4.1)	3518 (3.9)	299 (12.1)	<0.05
Cholesterol(mmol/L, mean(SD))	4.6 (1.0)	4.6 (1.0)	4.6 (1.0)	0.362
Triglycerides(mmol/L, mean(SD))	1.3 (0.6)	1.3 (0.6)	1.3 (0.6)	0.094
Lipoprotein A (mg/L, Median [Q1,Q3])	90.0 [43.0,182.0]	86.0 [41.0,176.0]	103.5 [47.0,196.2]	<0.05
Serum uric acid (umol/L,mean(SD))	312.1 (83.3)	312.5 (83.2)	303.2 (86.1)	<0.05
Creatinine(umol/L, mean(SD))	76.1 (14.2)	76.2 (14.1)	73.7 (15.4)	<0.05
Glycosylated hemoglobin (%, mean(SD))	5.8 (0.5)	5.8 (0.5)	5.9 (0.5)	<0.05
CEA (ng/ml, mean(SD))	2.0 (1.1)	2.0 (1.1)	2.2 (1.2)	<0.05
CA19-9 (IU/L, mean(SD))	10.6 (7.1)	10.6 (7.2)	10.9 (6.9)	0.158

### 3.3 Model performance evaluation

**Figure 4:**
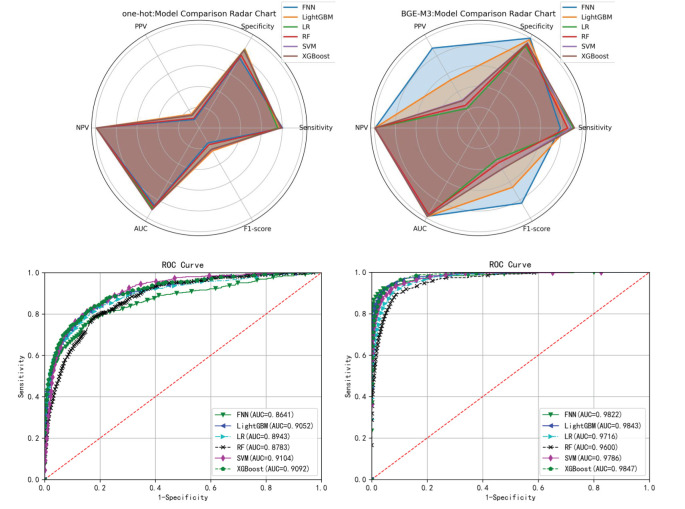
Radar plot and ROC curve of different models’ performance on the test set of the training cohort.

As shown in **Tables 3**, **4**, the BGE-M3 vectors achieved significant improvement compared to One-Hot vectors. Within the models using BGE-M3 vectors, XGBoost obtained the best performance in terms of AUC. As shown in **Figure 4**, the FNN obtain reasonable performance by using BGE-M3. These results demonstrated that the BGE-M3 vectors can show better performance on EMR data encoding than traditional One-Hot vectors.

**Table 3:** Performance of different models using one-hot vectors on the test set of training cohort.

Model	Sensitivity (mean ± std)	Specificity (mean ± std)	PPV (mean ± std)	NPV (mean ± std)	F1-score (mean ± std)	AUC (mean ± std)
FNN	0.8023 ± 0.0270	0.7796 ± 0.0293	0.0935 ± 0.0095	0.9929 ± 0.0008	0.1676 ± 0.0149	0.8641 ± 0.0118
LightGBM	0.7652 ± 0.0160	0.8799 ± 0.0036	0.1530 ± 0.0064	0.9925 ± 0.0005	0.2550 ± 0.0097	0.9052 ± 0.0067
LR	0.7573 ± 0.0161	0.8714 ± 0.0029	0.1430 ± 0.0033	0.9922 ± 0.0005	0.2406 ± 0.0052	0.8943 ± 0.0104
RF	0.7945 ± 0.0477	0.8094 ± 0.0182	0.1057 ± 0.0053	0.9929 ± 0.0013	0.1865 ± 0.0066	0.8783 ± 0.0085
SVM	0.7867 ± 0.0079	0.8659 ± 0.0042	0.1426 ± 0.0044	0.9931 ± 0.0003	0.2414 ± 0.0066	0.9104 ± 0.0090
XGBoost	0.7867 ± 0.0142	0.8623 ± 0.0028	0.1393 ± 0.0042	0.9930 ± 0.0004	0.2367 ± 0.0067	0.9092 ± 0.0064

**Table 4:** Performance of different models using BGE-M3 vectors on the test set of training cohort.

Model	Sensitivity (mean ± std)	Specificity (mean ± std)	PPV (mean ± std)	NPV (mean ± std)	F1-score (mean ± std)	AUC (mean ± std)
FNN	0.7895 ± 0.0455	0.9972 ± 0.0101	0.8864 ± 0.0217	0.9943 ± 0.0013	0.8351 ± 0.0199	0.9822 ± 0.0104
LightGBM	0.8603 ± 0.0104	0.9794 ± 0.0011	0.5339 ± 0.0018	0.9961 ± 0.0003	0.6589 ± 0.0021	0.9843 ± 0.0043
LR	0.9130 ± 0.0135	0.9101 ± 0.0033	0.2176 ± 0.0024	0.9974 ± 0.0004	0.3514 ± 0.0034	0.9716 ± 0.0025
RF	0.8603 ± 0.0219	0.9296 ± 0.0028	0.2507 ± 0.0042	0.9959 ± 0.0007	0.3883 ± 0.0069	0.9600 ± 0.0090
SVM	0.8927 ± 0.0113	0.9450 ± 0.0024	0.3077 ± 0.0026	0.9969 ± 0.0004	0.4577 ± 0.0040	0.9786 ± 0.0014
XGBoost	0.9251 ± 0.0047	0.9413 ± 0.0018	0.3015 ± 0.0074	0.9978 ± 0.0002	0.4547 ± 0.0079	0.9847 ± 0.0038

### 3.4 Independent test cohort validation

To assess the model’s generalization ability, this study employed 2023 data as an independent test cohort for validating the BGE-M3-based XGBoost model, which achieved the highest AUC on test set. The experimental results are summarized in **Table 5**, these results verified the robust generalization capability of our developed model.

**Table 5:** Results on the independent test cohort of the XGBoost model with the BGE-M3 vectors.

Model	Sensitivity	Specificity	PPV	NPV	F1-score	AUC
XGBoost	0.9253	0.9519	0.3306	0.9980	0.4872	0.9839

**Figure 5:**
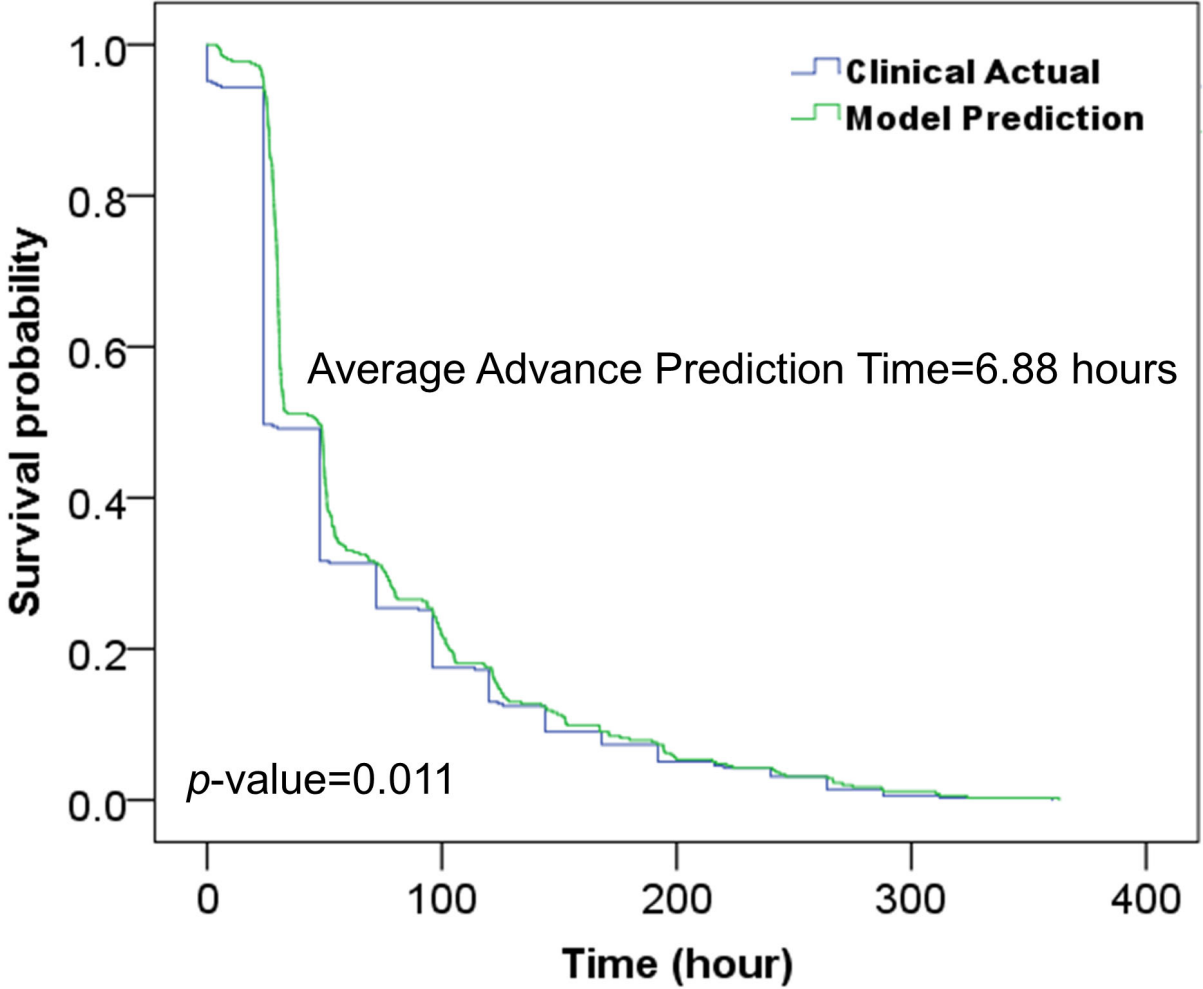
Kaplan-Meier analysis of the developed model and clinical actual diagnosis for evaluating the prediction time in advance.

This study evaluated the model’s ability to predict colorectal adenomas in advance using 431 hospitalized patients out of 482 positive samples from the independent test cohort. As shown in **Table 6**, the model achieved a prediction accuracy of 0.9141 for positive samples, with an average advance prediction time of 6.88 hours. Meanwhile, as shown in **Figure 5**, Kaplan-Meier analysis demonstrated that our proposed model predicted the high risk in advances (p<0.05) by an average of 6.88 hours compared to the actual clinical diagnosis time and maintained a reasonable accuracy.

**Table 6:** Average model prediction lead time.

Model	Correct predictions	Incorrect predictions	Predictive accuracy	Average advance prediction time (h)
XGBoost	394	37	0.9141	6.88

### 3.5 Assessment of model feature importance

**Figure 6:**
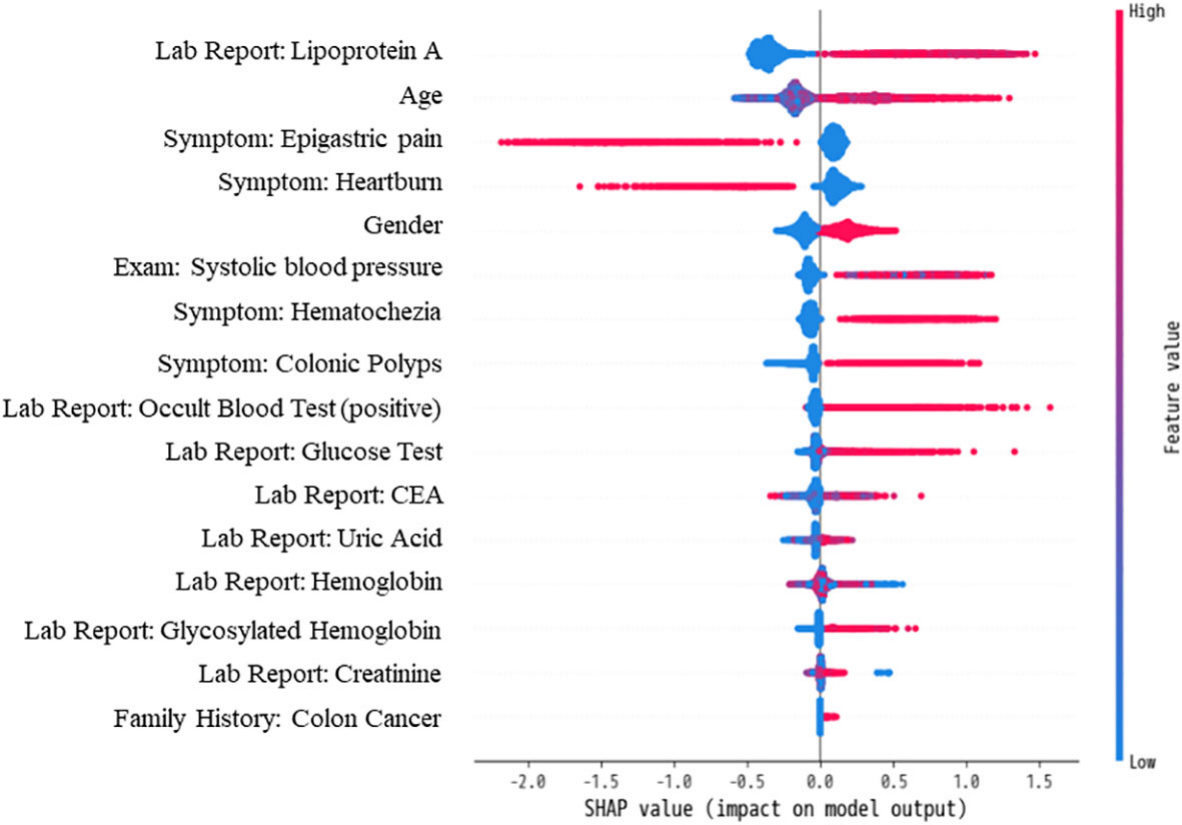
Contribution and directional influence of top 16 features on XGBoost Model predictions using SHAP analysis.

We utilized the SHAP method to interpret features’ contribution in the XGBoost model based on one-hot vectors. **Figure 6** illustrates the top 16 features ranked by SHAP importance, indicating their respective influence on model predictions. The vertical color gradient in **Figure 6** represents the magnitude of each feature’s SHAP value, with warmer colors indicating higher values and cooler colors indicating lower values. The horizontal SHAP values indicate whether each feature positively or negatively impacts predictions. Positive SHAP values support colorectal adenoma predictions, while negative values suggest non-colorectal adenoma predictions. Integrating the horizontal SHAP values with the vertical gradient provides a comprehensive understanding of the direction and strength of each feature’s influence on prediction outcomes.

**Figure 6** highlights the critical role of laboratory items in diagnosing colorectal adenoma, with eight out of the top 16 features belonging to this category. Notably, Lipoprotein A emerges as the most influential predictive feature. Additionally, symptomatic features prominently appear among the top 16, underscoring the importance of unstructured clinical EMR data in predictive modeling.

## 4 Discussion

This study successfully developed a highly accurate and generalizable risk prediction model for colorectal adenoma leveraging the strengths of LLM and comprehensive EMR data, providing a robust scientific foundation for early detection and intervention strategies. Furthermore, this study analyzed extensive clinical EMR of patients with colorectal adenoma, revealing distinct characteristics compared to non-colorectal adenoma patients.

Retrospective statistical analysis (**Table 2**) of the dataset identified typical clinical features associated with colorectal adenoma, including higher average age and male predominance, consistent with prior research (37–39). Symptoms such as blood in stool, abdominal pain, and stool abnormalities were prevalent among colorectal adenoma patients. Interestingly, nausea, less commonly associated with colorectal adenoma, exhibited significant inter-group differences, possibly reflecting variations in data collection across healthcare providers (40). Comorbidity analysis revealed significant associations with colon cancer, colon polyps, hypertension, and family history of diabetes mellitus, aligning with existing literature (41–44). The analysis also highlighted variations in biomarkers including hemoglobin, fecal occult blood test, lipoprotein A, serum uric acid, creatinine, glycated hemoglobin, and carcinoembryonic antigen (CEA) (45–49), underscoring their relevance in predicting colorectal adenoma risk.

The comparison (**Table 3**, **4**) between BGE-M3 and one-hot vector coding demonstrated the superior performance of BGE-M3 dense representation, dimensional reduction, semantic information capture, context sensitivity, and adaptive learning. This enhanced the model’s ability to handle complex clinical EMR data, resulting in improved performance across various machine learning algorithms. XGBoost emerged as the top performer, demonstrating robust sensitivity and AUC values of 0.9251, 0.9413, and 0.9847, respectively, leveraging advantages in boosting framework, feature engineering, regularization, pruning, and parallel computing (33).

The model’s generalizability was validated on an independent test cohort (**Table 5**), utilizing the BGE-M3 semantic vector model and diverse data sources from several departments (**Figure 3**) (50). Our model facilitated earlier detection of colorectal adenoma (**Table 5**, **Figure 5**), providing a potential opportunity to optimize clinical workflows and improve patient outcomes. SHAP analysis (**Figure 6**) identified key predictors such as age, symptoms, family history, biomarkers, and cardiovascular risk factors (51). Notably, examination-related features did not appear in the TOP16 list, which may be attributed to two factors: (1) The study focused on early-stage prediction of colorectal adenomas in patients with subtle symptoms that might not manifest distinctly in imaging data; (2) To ensure objectivity, the research protocol specifically excluded examination methods directly associated with pathological confirmation (including but not limited to colonoscopy, histopathological analysis, and tissue biopsy). This lack of significant contribution from imaging features compared to other predictors could therefore be explained by the inherent limitations of early disease detection and the deliberate exclusion of gold-standard diagnostic procedures.

Our exclusion of patients with a history of colectomy or conditions potentially affecting inflammation assessment is methodologically appropriate and preserves the generalizability of the model within its intended screening population. These individuals are excluded because their underlying conditions (e.g., post-colectomy anatomical alterations, inflammatory comorbidities) may confound adenoma-specific biomarker profiles critical for early-stage colorectal cancer screening (52–55). This exclusion aligns with standard clinical practice, where such patients are typically diverted from routine adenoma screening protocols and instead undergo dedicated surveillance pathways tailored to their specific risk profiles (56–58). Consequently, the exclusion criteria do not compromise the model’s applicability to its target population—asymptomatic individuals eligible for primary screening.

However, it is important to acknowledge the limitations of this study, which do not affect the conclusions of this study. Firstly, the single-center nature of the study may limit the generalizability of the results to diverse healthcare settings. While multi-center validation was precluded by privacy regulations governing EMR sharing, we implemented prospective temporal validation (isolated 2023 test cohort) to approximate external generalizability, ensuring no data leakage between training (2012–2022) and testing phases. Secondly, the retrospective design does not allow for direct assessment of the model’s impact on clinical practice. Future research should focus on integrating the model into clinical support systems for prospective validation.

Furthermore, practical challenges persist in translating this model into clinical practice: (1) Workflow Integration Framework: To operationalize the 6.88-hour predictive lead time, a dual-phase implementation strategy is proposed. First, standardized application programming interfaces (APIs) will bridge heterogeneous EMR systems, generating context-aware alerts (e.g., prioritized task flags or pop-up notifications) for high-risk patients. Second, an interpretable clinician dashboard will dynamically display risk stratification, highlighting SHAP-identified predictors (e.g., lipoprotein A >100 mg/L) and evidence-based escalation protocols (e.g., “urgent colonoscopy ≤48 hours”) to align with clinical workflows without imposing cognitive burdens. (2) Strategic Value of Temporal Advantage: The 6.88-hour window holds distinct clinical implications across care settings. In acute care, it enables time-sensitive interventions (e.g., accelerating endoscopic evaluation for occult bleeding), mitigating risks of obstruction or hemorrhagic complications. For ambulatory care, it streamlines triage prioritization, reducing diagnostic latency through early risk stratification. (3) Adoption Barrier Mitigation: To overcome implementation inertia, three evidence-driven solutions are prioritized: (i) Prospective multicenter trials evaluating clinical endpoints (adenoma detection rate, interval-to-diagnosis); (ii) Adaptive alert thresholds informed by clinician feedback (e.g., muting redundant alerts for patients under diagnostic workup); (iii) Privacy-preserving federated learning architectures to enhance cross-institutional generalizability. Standardized validation protocols are being finalized to ensure translational fidelity across diverse healthcare ecosystems.

## 5 Conclusion

This study presents a novel approach to colorectal adenoma risk prediction leveraging clinical EMR data and LLM. The proposed model demonstrates superior performance in identifying high-risk individuals, providing a valuable tool for early screening. By leveraging the BGE-M3 semantic vector model, the algorithm enhances data comprehension and captures nuanced semantic information. With an average lead time of 6.88 hours and outstanding classification performance, the model has the potential to revolutionize clinical workflows and improve patient care.

## Data Availability

The original contributions presented in the study are included in the article/**Supplementary Material**. Further inquiries can be directed to the corresponding author.
