# Supplementary material for: Early prediction of colorectal adenoma risk: leveraging large-language model for clinical electronic medical record data
Source: Front Oncol. 2025 May 15;15:1508455. doi: 10.3389/fonc.2025.1508455 (PMC12119310; doi:10.3389/fonc.2025.1508455)
Supplement: Supplementary file 1 [file DataSheet1.docx]

Early Prediction of Colorectal Adenoma Risk: Leveraging Large-Language Model for Clinical Electronic Medical Record Data - Supplementary Material

# 2 MATERIALS AND METHODS

## 2.4 Details of Model Training

FNN is an algorithmic model that mimics the neuronal networks found in the human brain, using neurons with nonlinear activation functions as basic units. By connecting numerous neurons across multiple layers, neural network models can effectively address a wide range of complex problems. Error backpropagation algorithms are typically employed to iteratively update and optimize model parameters. The advancement of deep learning technology in recent years has led to the widespread adoption of various neural network models across diverse fields. The primary architecture used in this study features a shallow neural network structure with 2 hidden layers in addition to the input and output layers.

LR is one of the simplest machine learning algorithms, utilizing linear regression and sigmoid activation functions for predicting outcomes in binary classification tasks. The logistic regression model not only predicts categories but also estimates probabilities, approximating the log odds of true labels. Despite its simplicity and speed, logistic regression performs inadequately on complex nonlinear tasks. The formula for the probability of a binary logistic regression model with a label value of 1 is:

$P\left( Y=1|x \right)=\frac{exp(wx)}{1+exp(wx)}$ (1)

RF is a straightforward classification model that employs the Bagging technique. It randomly selects subsets of data and features to train multiple weak classifiers (decision trees), whose collective predictions are determined through voting. RF effectively mitigates overfitting to improve prediction accuracy.

SVM is a high-performance machine learning algorithm classified into linearly separable SVM, linear SVM, and nonlinear SVM. By employing kernel functions, SVM maps data into high-dimensional spaces, enabling the construction of hyperplanes that effectively separate nonlinear data points. SVM demonstrates robustness and high generalization across various tasks. The classification decision function of SVM is:

$f\left( x \right)=sign(\sum_{i=1}^{N} a_{i}y_{i}exp(-\frac{||x-z{||}^{2}}{2б^{2}}+b))$ (2)

where *sign* denotes the sign function, $a_{i}$ represents the Lagrange multiplier, $y_{i}$ denotes the data label, *z* denotes the data mean, *б* denotes the standard deviation, and *b* represents the bias term.

LightGBM and XGBoost are tree-based boosting algorithms. LightGBM employs a histogram algorithm to discretize continuous feature values into n integer bins. During data traversal, statistics are accumulated within these bins, optimizing segmentation points for subsequent data passes. This approach significantly reduces memory and time consumption compared to full dataset traversal. LightGBM also utilizes techniques such as gradient-based one-sided sampling and feature bundling to further enhance algorithm efficiency and reduce memory footprint.

In contrast, XGBoost is a gradient boosting decision tree algorithm that iteratively fits residuals of the previous model iteration rather than actual labels. It approximates these residuals using the negative gradient of the loss function, incorporating second-order Taylor expansion and regularization to prevent overfitting and accelerate convergence on datasets. The objective function of XGBoost is formulated as:

$f=\sum_{i=1}^{n} l\left( y_{i},\hat{y}_{i} \right)+\sum_{k=1}^{K} Ω(f_{k})$ (3)

where $l$ is the loss function, $y_{i}$ are the actual labels, $\hat{y}_{i}$ are the predicted values, $Ω$ denotes the regularization term, and $f_{k}$ represents the individual trees in the ensemble.

### 2.4.1 Optimization of Model Parameters

To ensure a fair comparison among the models, the parameters of each algorithm were systematically optimized using grid search and five-fold cross-validation. The optimal parameters and the range of parameters considered for grid search are detailed in Table S1.

**Table S1** Optimal parameters and the details of parameter range for searching

| Model | Optimal Parameters | Meaning | Parameter Range | Parameter Tuning Step |
| --- | --- | --- | --- | --- |
| FNN | nb_epoch=500 | Iterations | 100-1000 | 100 |
|  | drop_out=0.3 | Feature Dropout Rate | [0.1,0.3,0.5] |  |
|  | units=128 | Output Dimension | [128] |  |
|  | activation='relu' | Activation Function | ['relu', 'linear'] |  |
|  | dense_unit=[256,512] | Hidden Layer Parameters | [128,256], [256,256], [256,512] |  |
| LightGBM | learning rate=0.08 | Learning Rate | [0.001,0.005,0.01,0.05,0.08,0.1] |  |
|  | max_depth=6 | Maximum Tree Depth | 5-10 | 1 |
|  | colsample_bytree=0.8 | Subsample Ratio of Columns | 0.8-1.0 | 0.05 |
|  | min_child_samples=5 | Minimum Data Points for a Split | 5-25 | 5 |
|  | n_estimators=300 | Number of Trees | 100-500 | 100 |
|  | reg_alpha=0.1 | L1 Regularization Penalty | 0-0.1 | 0.02 |
|  | reg_lambda=0.1 | L2 Regularization Penalty | 0-0.1 | 0.02 |
|  | subsample=1 | Training Sample Ratio | 0.8-1 | 0.05 |
| LR | penalty='l2' | Regularization Penalty Type | ['l1', 'l2', 'elasticnet'] |  |
|  | solver='liblinear' | Optimizer Type | ['liblinear', 'sag'] |  |
| RF | n_estimators=500 | Number of Trees | 100-500 | 100 |
|  | max_depth=15 | Maximum Tree Depth | [3,7,12,15] |  |
|  | min_samples_split=10 | Minimum Samples Required to Split a Node | [10,20,50,100] |  |
|  | min_samples_leaf=3 | Minimum Samples Required at a Leaf Node | [3,5,10,15,20] |  |
|  | max_features=30 | Number of Features Used for Best Split | [7,10,15,20,30] |  |
| SVM | C=1 | Regularization Penalty Coefficient | 0-2 | 0.5 |
|  | kernel='rbf' | Kernel Function Type | ['linear', 'poly', 'rbf', 'sigmoid'] |  |
| XGBoost | learning rate=0.05 | Learning Rate | [0.001,0.005,0.01,0.05,0.08,0.1] |  |
|  | max_depth=8 | Maximum Tree Depth | 5-10 | 1 |
|  | colsample_bytree = 0.8 | Subsample Ratio of Columns | 0.8-1.0 | 0.05 |
|  | gamma=0 | Minimum Loss Function Value | 0-2 | 0.5 |
|  | n_estimators=300 | Number of Trees | 100-500 | 100 |
|  | reg_lambda=1 | L2 Regularization Penalty | 0-0.1 | 0.02 |
|  | subsample=1 | Training Sample Ratio | 0.8-1 | 0.05 |

## 2.5 Details of Evaluation Metrics

The performance of the risk prediction model was evaluated using multiple metrics in both the validation cohort and the independent test cohort, Sensitivity/Recall, Specificity, Positive Predictive Value (PPV), Negative Predictive Value (NPV), and F1-Score. The calculation formulas for each metric are as follows:

For binary classification, the actual and predicted labels can be represented in a matrix form, as shown below:

**Table S2** Confusion Matrix

|  | Predicted Label | |
| --- | --- | --- |
| Actual Label | Colorectal Adenoma | Non-Colorectal Adenoma |
| Colorectal Adenoma | TP | FN |
| Non-Colorectal Adenoma | FP | TN |

Sensitivity/Recall, also known as the true positive rate, is the percentage of actual patients correctly identified as such by the model. It reflects the model's ability to detect patients with the disease.

$Sensitivity/Recall=\frac{TP}{TP+FN}$ (4)

Specificity, also known as the true negative rate, is the percentage of individuals who are not diseased and are correctly identified as not having the disease by the model. It reflects the model's ability to correctly identify non-patients.

$\mathrm{Spe}cificity=\frac{\mathrm{TN}}{TN+FP}$ (5)

PPV is the percentage of patients predicted by the model to have the disease who are actually diseased.

$PPV=\frac{\mathrm{TP}}{TP+FP}$ (6)

NPV is the percentage of individuals predicted by the model to be disease-free who are indeed not diseased.

$NPV=\frac{TN}{TN+FN}$ (7)

F1-Score provides a harmonic mean of Precision and Recall, balancing the two metrics.

$F1-Score=\frac{2\cdot Precision\cdot Recall}{Precision+Recall}$ (8)
